# In search of an evidence-based strategy for quality assessment of human tissue samples: report of the tissue Biospecimen Research Working Group of the Spanish Biobank Network

**DOI:** 10.1186/s12967-019-2124-8

**Published:** 2019-11-12

**Authors:** Margalida Esteva-Socias, María-Jesús Artiga, Olga Bahamonde, Oihana Belar, Raquel Bermudo, Erika Castro, Teresa Escámez, Máximo Fraga, Laura Jauregui-Mosquera, Isabel Novoa, Lorena Peiró-Chova, Juan-David Rejón, María Ruiz-Miró, Paula Vieiro-Balo, Virginia Villar-Campo, Sandra Zazo, Alberto Rábano, Cristina Villena

**Affiliations:** 1 Centro de Investigación Biomédica en Red Respiratory Diseases (CIBERES), Plataforma Biobanco Pulmonar CIBERES, Hospital Universitari Son Espases, Palma, Spain; 2 Grupo de Inflamación, reparación y cáncer en enfermedades respiratorias, Institut d’Investigació Sanitària de les Illes Balears (IdISBa), Hospital Universitari Son Espases, Palma, Spain; 3 CNIO Biobank, Spanish National Cancer Centre (CNIO), Madrid, Spain; 4 INCLIVA Biobank, Valencia, Spain; 5 Basque Foundation for Health Innovation and Research, Basque Biobank, Barakaldo, Spain; 6 Hospital Clínic-IDIBAPS Biobank, Institut d’Investigacions Biomèdiques August Pi i Sunyer (IDIBAPS), Barcelona, Spain; 7 IMIB Biobank, Instituto Murciano de Investigación Biosanitaria, Murcia, Spain; 8 Depto. de Ciencias Forenses, Anatomía Patolóxica, Xinecología e Obstetricia, e Pediatría, Facultade de Medicina, Universidade de Santiago de Compostela (USC), Santiago, Spain; 9 Biobanco Complejo Hospitalario Universitario de Santiago de Compostela (CHUS), SERGAS, Santiago, Spain; 10 University of Navarra’s Biobank-IdiSNA, Pamplona, Spain; 11 Vall d’Hebron University Hospital Biobank, Vall d’Hebron Hospital Research Institute, Barcelona, Spain; 12 Biobanco del Sistema Sanitario Público de Andalucía, Granada, Spain; 13 IRBLleida Biobank, Instituto de Investigaciones Biomédica de Lleida-Fundación Dr. Pifarre, Lérida, Spain; 14 Department of Pathology, IIS-Fundación Jiménez Díaz, Madrid, Spain; 15 Banco de Tejidos, Fundación CIEN, Instituto de Salud Carlos III, Madrid, Spain

**Keywords:** Quality, Pre-analytical variables, Biobank, Tissue, Biospecimen science

## Abstract

The purpose of the present work is to underline the importance of obtaining a standardized procedure to ensure and evaluate both clinical and research usability of human tissue samples. The study, which was carried out by the Biospecimen Science Working Group of the Spanish Biobank Network, is based on a general overview of the current situation about quality assurance in human tissue biospecimens. It was conducted an exhaustive review of the analytical techniques used to evaluate the quality of human tissue samples over the past 30 years, as well as their reference values if they were published, and classified them according to the biomolecules evaluated: (i) DNA, (ii) RNA, and (iii) soluble or/and fixed proteins for immunochemistry. More than 130 publications released between 1989 and 2019 were analysed, most of them reporting results focused on the analysis of tumour and biopsy samples. A quality assessment proposal with an algorithm has been developed for both frozen tissue samples and formalin-fixed paraffin-embedded (FFPE) samples, according to the expected quality of sample based on the available pre-analytical information and the experience of the participants in the Working Group. The high heterogeneity of human tissue samples and the wide number of pre-analytic factors associated to quality of samples makes it very difficult to harmonize the quality criteria. However, the proposed method to assess human tissue sample integrity and antigenicity will not only help to evaluate whether stored human tissue samples fit for the purpose of biomarker development, but will also allow to perform further studies, such as assessing the impact of different pre-analytical factors on very well characterized samples or evaluating the readjustment of tissue sample collection, processing and storing procedures. By ensuring the quality of the samples used on research, the reproducibility of scientific results will be guaranteed.

## Background

Human tissue samples obtained from biopsies, surgical specimens, organ transplants and autopsies are a great resource to find potential targets to aid clinical decisions such as diagnosis and treatment of diseases. Over the last decades, the use of human biospecimens has heavily increased in biomedical research in order to evaluate the outcome, survival, and new therapies for patients, and also to test new hypotheses related to the genetic and molecular basis of diseases. Besides, the constant technology advances for biomarker discovery have led to an increasing demand of large sets of human biospecimens and for new formats for the preservation of biospecimens suitable for these technologies, promoting also the creation of new human biorepositories [1].

Research on disease biomarkers is one of the main requirements for the progress of personalized medicine and its use for targeted therapies [2–5]. This clinical approach, particularly in Oncology, allows a great number of patients to access more efficient and safer therapeutic protocols, which have been selected according to molecular findings in tissue samples obtained from patients for diagnostic or therapeutic purposes. Indeed, many studies report the sustained discovery of different clinical biomarkers with potential application to personalized medicine [6]. However, most of them cannot be applied to clinical practice due to a lack of high sensitivity or/and specificity, compromising its reproducibility and its successful clinical implementation [7]. In this context, the recruitment of subjects, as well as the selection and management of tissues, is critical in biomarker research [8, 9].

Specifically, it is well known that the handling of human biospecimens during their collection, processing and storage can alter their characteristics and influence their quality, integrity and/or molecular composition [10]. These variations are considered as a bias in biomarker discovery, hindering the development of new targeted therapies.

As a result, there is a crucial need for the standardization of collection, processing and storage procedures to improve the quality of biospecimens, in order to enhance the reproducibility of biomarker development. Consequently, in recent years, a large number of strategies have been described to standardize and improve the quality control of human samples for their use in biomedical research, such as the “Standard PREanalytical Code” (SPREC) version 2.0, a method developed and agreed by the International Society for Biological and Environmental Repositories Biospecimen Science Working Group, which allows controlling the main pre-analytical factors that may have an impact on the integrity of the biological sample during its collection, processing and storage. SPREC assigns to each sample a code of 7 elements based on its pre-analytical characterization, helping to standardize the quality of the set of samples to be used [11].

There are also some guidelines to guarantee collection of clinical and pre-analytical data, such as BRISQ (Biospecimen Reporting for Improved Study Quality [12]. The aim of BRISQ is to ensure the registration of human samples data, including the preanalytical factors which could influence the integrity, quality or molecular composition such as (a) type of pathology, (b) clinical status and features of the patient and (c) handling and preservation conditions (for example: stabilization, shipping and storage conditions).

However, despite the efforts made in the last years, the scientific community still lacks a standardized approach to ensure and evaluate the clinical and research usability of human tissue samples. For these reasons, we have undertaken to summarize and give a general overview of the current situation concerning quality assurance in human tissue biospecimens.

## Literature review

The Spanish Biobank Network (SBN), formed by 39 biobanks, provides mainly samples to the scientific community to support biomedical research, as well as technical, ethical and legal advice, and other services related to human biological samples. One of its most developed areas is the Biospecimen Science, where mainly biologists and pathologists from 13 biobanks of the SBN, participate cooperatively in a working group focused on innovation in human tissues handling, (i) for improving standards on tissue collection, processing and storage, and (ii) for setting a global quality assessment method of human tissues for biomedical research.

Firstly, the SBN Biospecimen Science working group conducted an exhaustive literature review of the analytical techniques used to evaluate the quality of human tissue samples over the past 30 years, as well as their reference values if they were published, and classified them according to the biomolecules evaluated: (i) DNA, (ii) RNA, and (iii) soluble or/and fixed proteins for immunochemistry. The group focused on publications where colon, breast, kidney, lung, ovary or brain tissues were used, since these organs are the main source of samples collected by the participating biobanks.

Secondly, based on results of the literature review and the expertise of the participating biobanks, a proposal for quality assessment of tissues based on the type of preservation method and biomolecule of interest was led. The algorithm was purposed to classify the solid tissue samples according their expected quality, taking into account the type of analytical technique required for the research project.

For the design of the algorithm, the Group made a prioritization of pre-analytical factors defined by SPREC v.2.0 [11] and BRISQ [12] with the highest expected impact on the integrity of tissue samples according to the literature. So, the Working Group classified in three categories (optimal or non-effect, moderate or unknown effect and suboptimal quality) the expected quality of the nucleic acids integrity and fixed proteins for immunochemistry for each factor, both in snap-frozen and in formalin fixed-paraffin embedded (FFPE) tissue samples.

## Identification of techniques for tissue quality assessment

More than 130 publications released between 1989 and 2019 were analysed, most of them reporting results based on the analysis of tumour and samples from biopsy procedures. Tables 1, 2, 3, 4 show a summary of the analytical techniques used to evaluate tissue quality, according to the analysed biomolecule (RNA, DNA, soluble proteins and antigenicity, respectively). They describe (i) the measurement method of the biomolecule, (ii) the analytical technique used, (iii) the parameters of the evaluated biomolecule, (iv) the threshold values and (v) the anatomical organ analysed.

**Table 1:** Summary of publications evaluating quality of RNA samples

Measurement method	Analytical technique	Evaluated parameter	Threshold	Organ	References
Spectrophotometry	UV spectroscopy (A260/280) NanoDrop	Quantity and purity	Around 2	Human trabecular bone	[13–18]
			> 1.8 excellent	Colon, articular cartilage and subchondral bone, brain	
			1.8–1.6 adequate		
			< 1.6 inadequate		
	UV spectroscopy (A260/230) NanoDrop	Quantity and purity	> 2 non contaminated RNA	Articular cartilage and subchondral bone	[16]
			< 2 contaminated RNA		
Electrophoresis	RIN, RIS, or equivalent	Integrity	≥ 7 high-integrity RNA	Colon, kidney, placenta, articular cartilage and subchondral bone, trabecular bone, pancreas	[13, 14, 16, 19–27]
			6–7 adequate-integrity RNA	Trabecular bone, pancreatic, stomach, liver, colon, brain	[13, 26, 28–33]
			5–6 low integrity	Pancreas, breast, thyroid, stomach, lung, colon	[26, 34, 35]
			3–5 partially degraded	Breast, thyroid, stomach, lung, colon, kidney, pancreas	[14, 26, 33–35]
			1–3 totally degraded	Trabecular bone, breast, thyroid, stomach, lung, colon, brain, placental	[13, 17, 27, 34, 36]
	DV200	Integrity	> 70% high quality	Brain and other tissue types	[37, 38]
			50–70% medium quality		
			30–50% low quality		
			< 30% too degraded		
	28S:18S peak ratio	Integrity	Around 2	Stomach, pancreas, liver, colorectal	[29]
	Electrophoretic profile	Integrity	2 bands 2000 nt (18S), 4000 nt (28S) → (Non-degraded RNA)	Pancreatic tissue	[26, 27]
			Diffuse banding indicative of degraded RNA	Pancreatic tissue	[26, 27]
RT-qPCR	3′:5′ ratio	Integrity	1–5 perfectly intact mRNA	N/A	[39]
			> 5 suggests degradation		
			≥ 10 denatured mRNA		
	Ct values	Functionality	Increasing Ct values of *ABL1*, *FOSB* and *JUN* genes suggest RNA degradation	Colon	[40]

**Table 2:** Summary of publications evaluating quality of DNA samples

Measurement method	Analytical technique	Evaluated parameter	Quality stratification threshold	Organ	References
Spectrophotometry	UV spectroscopy (A260) NanoDrop	Quantity	–	Pancreas, spleen, duodem, liver	[41]
	Fluorochrome binding and fluorometer (Qubit)	Quantity	**–**	Pancreas, spleen, duodem, liver, sarcoma, breast, gastric, colorectal, prostate, lung adenocarcinoma	[41–45]
	UV spectroscopy (A260/280) NanoDrop	Purity	1.8–2.1 optimal, < 1.8 or > 2.1 contamination with RNA proteins or others	Lung adenocarcinoma, prostate	[44, 45]
	UV spectroscopy (A260/230) NanoDrop	Purity	2–2.2 optimal, lower ratios may indicate presence of contaminants	Prostate	[44]
Electrophoresis	Pulsed field gel electrophoresis	Fragmentation	Size distribution between 12 and 300 kb	–	[46]
	Agarose gel, and capillary electrophoresis (DNA Integrity Number, DIN)	Fragmentation		Pancreas, spleen, duodenum, liver, sarcoma	[41, 47, 48]
PCR	Multiplex PCR and dHPLC/multiplex PCR and gel electrophoresis	Functionality	Presence of the 300- to 400-bp amplicon indicates optimal quality, amplicon sizes ranging from 102 to 300 bp	Brain, colon and prostate	[44, 49]
	Multiplex PCR and gel electrophoresis	Functionality	Threshold not defined (amplicons between 268 and 1327 bp), optimal samples with amplification of 200 bp fragment or larger	Colon, uterine, myometrium and liver, breast	[50, 51]
	Multiplex PCR and microfluidic analysis	Functionality	A QC ratio above 0.20 indicates optimal quality, ratios below 0.20 suggests moderate or poor quality	Lung	[52]
	Multiplex digital PCR (dPCR)	Functionality	Validation needed to establish stratification thresholds	Lung	[53]
	qPCR	Functionality	Increasing qPCR ratio between frozen and FFPE tissue samples, 93 bp human GAPDH qPCR, detection of 18S5 rRNA by qPCR (CT-value < 38), qPCR using FFPE QC kit and PreSeq QC assay, Q-ratio (with a value between 0 and 1), in which 41 bp and 129 bp targets were amplified by qPCR (KAPA human genomic DNA quantification and QC Kit-KAPA Biosystems). High Q-ratio: less fragmentation and vice versa	Liver, breast, tongue, prostate, sarcoma, lung adenocarcinoma, breast, gastric, colorectal	[43, 45, 47, 54, 55]
	Multiplex qPCR	Functionality	percentage of functional templates (QFI, ranging from 0.03 to 24.5%), optimal > 3% to 6%	Different sources	[56]

MF (somagen diagnostics) is a mixture of methanol and polyethylene glycol (90% and 10%, respectively)

**Table 3:** Summary of publications of quality control tools used in proteomics for evaluating the impact of pre-analytical factors

Measurement method	Analytical technique	Evaluated parameter	Pre-analytical factor	Threshold	Organ	References
Spectrophotometry	DC protein assay	Concentration determined based on standard curve	N/A	N/A	Colon, kidney	[57, 58]
	BCA protein assay					
Electrophoresis	Western blot	PCNA detection	Fixation	–	Colon	[57]
		Comparative evaluation of reactivity of fresh and FFPE using antibodies against GAPDH, tropomyosin, vinculin and myosin	–	–	Sheep tissue from skeletal muscle, liver, human hyperplastic thyroid tissue	[59]
	SDS-PAGE and silver staining	Size distribution	Sample age	High quality proteins are feasible to extract from 14 years samples	Liver	[60]
		*N*-cadherin and phospo-ERK detection				
	2D-PAGE	Comparison of 2D-PAGE gel protein profiles	Time to freeze	30 min	Kidney	[61]
	Immunoblotting	P-p27 detection	–	–	Cell culture	[62]
Mass spectrometry	LC–MS/MS analysis	Comparative analysis of peptide hits between fresh-frozen and FFPE samples	Fixation	–	Muscle	[59]
		Protein overlap between fresh and FFPE tissue sections	Fixation	–	Kidney	[58]
	Capillary isoelectric focusing coupled with RP LC–MS/MS	–	Storage time	From 7 years fewer distinct peptides and proteins were identified but the normalised expression values of actin, desmin and progesterone receptor were consistent until 12 years	Mesenchyme	[63]
Protein microarray	RPPAs	Evaluation of increase and decrease percentage of phosphoproteins	Time to fixation	20 min	Uterus, colon, lung, ovary, breast, lymph node	[64]

**Table 4:** Summary of publications evaluating antigenicity quality

Analytical technique	Evaluated parameter	Pre-analytical factor	Threshold	Organ	References
Quantitative IF (AQUA score)	ER, HER2, Ki-67, CK	Storage time	IF signal decreases 10% in 4–8 years depending on the marker	Breast	[65]
	Increased marker: 95th percentile of slope for n = M is higher than 0	CIT	Labile and loss of antigenicity within 1–2 h of CIT	Breast	[66]
	Decreased marker: 95th percentile of slope for n = M is lower than 0				
	No changes in marker: 95% CI for the slope with both n = M and n = 10 × M Including the zero slope				
	Trend up/trend down: 95% CI for the slope with n = 10 × M not including it				
	Cytokeratin, pERK1/2 and pHSP-27 expression	CIT	Negative TQI values (as indicator of loss of tissue quality) for increasing CIT	Breast	[67]
IHC	Vimentin	Fixation	–	Melanoma	[68]
	ER and PgR	Fixation, slicing, storage of slides	Samples for ER and PgR testing are fixed in 10% NBF for 6 to 72 h. CIT < 1 h. Samples should be sliced at 5-mm intervals. Storage of slides for more than 6 weeks before analysis is not recommended	Breast	[69]
	P-p27	–	–	–	[70]
	SNRPA and SnRNP70 H-score	Fixation	H-score < 60 as a cut off for positive signal	Breast	[71]
	MAP2	Fixation, slicing and storage	Decrease of MAP2 immunoreactivity in unfixed and in delayed-fixed	Rat brain	[72]
	Actin, desmin and progesterone receptor staining	Storage time	Consistent staining over 18 years	Mesenchyme	[63]

It should be mentioned that the Group found little information focused on quality control of soluble proteins (Table 3) and antigenicity, including objective threshold values and analytical techniques used (Table 4). For this reason, the Working Group decided to include the most relevant publications regarding pre-analytical factors and its consequent effect on them. Consequently, Tables 3 and 4 include information regarding the pre-analytical factor under study for each cited reference and, if known, the threshold established to determine the effect of the pre-analytical factor on the sample.

## Consensus on an integrated algorithm for quality assessment

With the aim of systematizing the classification of human tissue samples according to their expected quality, a categorization proposal has been drawn up in the present study, based on SPREC and BRISQ tools as reference. Becker et al. [73] has already eloquently discussed in a review paper the importance of these pre-analytical factors for the meaningful translation of proteomic methods and findings to clinical practice. Next, in order to verify the functionality of the proposed categories and to establish reference ranges of analytical values, an algorithm was designed for decision-making based on the different biomolecules with different susceptibility profiles and on the type of sample preservation.

## A quality assessment proposal for frozen tissue samples

**Fig. 1:**
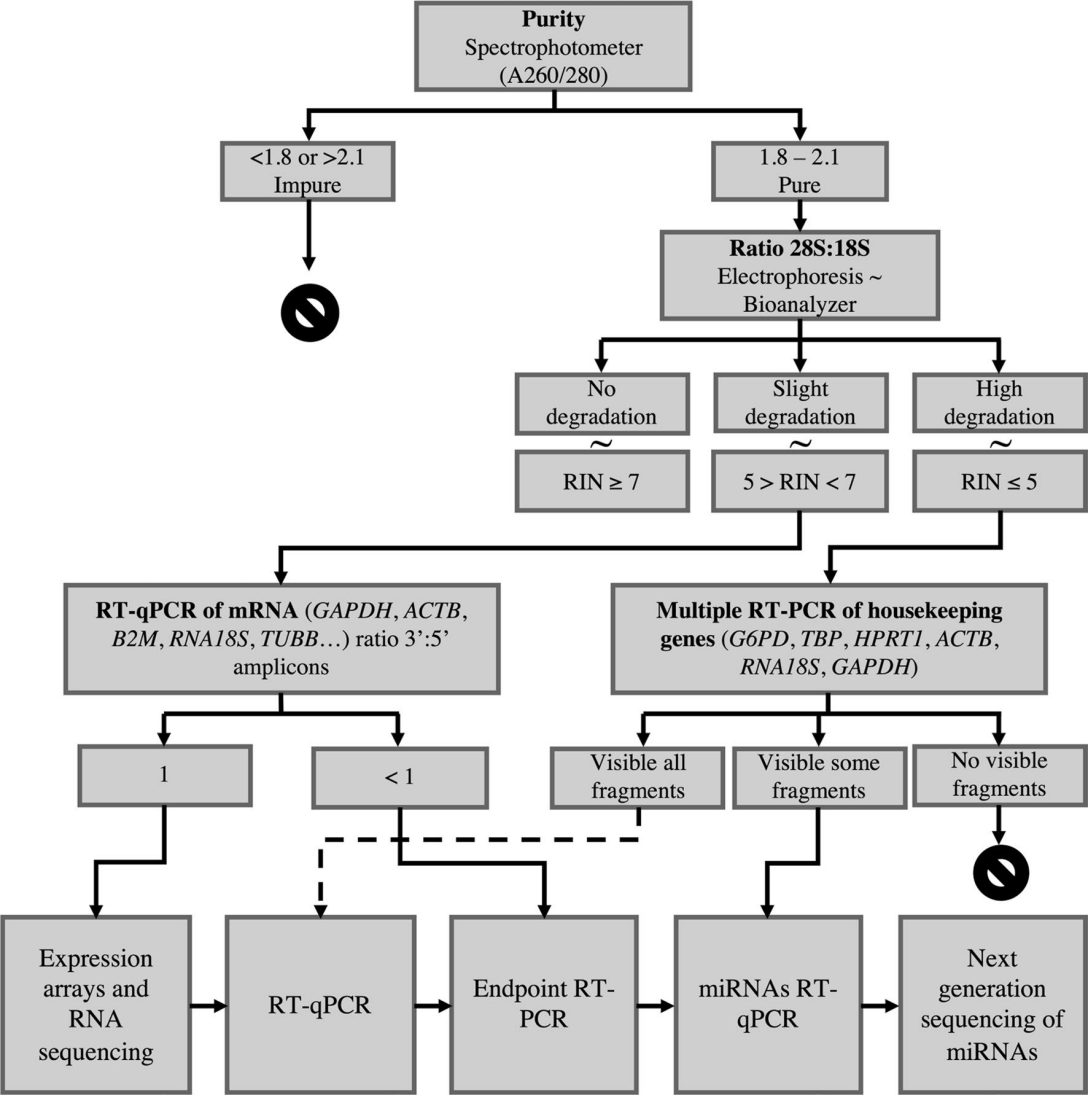
Procedures proposed to evaluate molecular integrity in order to classify the suitability of samples for expected applications

Because of the increasing use of human frozen tissue specimens as a gold-standard for molecular analysis, a testing approach was designed for frozen tissue samples based on RNA evaluation (Fig. 1). As a first step, purity and concentration assessment of total RNA through spectrophotometry is recommended, since it is a quick and relatively simple method to evaluate (1) great deteriorations according to SPREC variables suffered during storage or analysis, or (2) a low cellular content related to its anatomical origin.

In case that an adequate concentration of total RNA is obtained and it is necessary to evaluate the suitability of the sample to perform gene expression studies, it would be advisable to evaluate the potential effect of pre-analytical factors (SPREC and BRISQ) on RNA integrity (Table 5) to decide if further optional analyses are required to determine whether a sample is suitable to the research purpose.

**Table 5:** Expected quality for frozen tissue samples based on RNA quality assessment according to pre-analytical factors prioritized following SPREC and BRISQ recommendations

Type of codification	Variable	Optimal expected quality	Moderate expected quality	Sub-optimal expected quality
BRISQ	Anatomical site	Colon, lung and liver	Fatty	–
			Fibrous	
			Pancreatic	
			Neuronal [74–76]	
BRISQ	Body temperature	4 °C (post-mortem) [77]	RT 18–28 °C (post-mortem) [77]	Unknown
		37 °C (alive)		
SPREC	Type of sample	CEN, CLN, FNA, LCM, PLC, TIS	HAR, PEN, TCM	ZZZ
SPREC	Type of collection	A06, BCM, BPS, BSL, BTM, FNA, PUN, SRG, SSL, STM, VAC	A12	A24, A48, A72 [77]
				SWB
SPREC	Warm ischemia time	A, B, C, D, N	E	F, X
SPREC	Cold ischemia time	A, B, C, D [20, 78]	E, N	F, X [30, 66, 79]
SPREC	Fixation/stabilization type	OCT, PXT [80, 81]	None	Others (ACA, ALD, FOR, HST, NAA, NBF, XXX, ZZZ) [83]
		RNL [32, 82]		
		SNP [24, 80, 81]		
		ALL		
SPREC	Fixation/stabilization time	D, E (PXT) [84]	A, B, C	X
		F (ALL, RNL) [85]	D, E (ALL, RNL) [85]	
		N	F, G (PTX) [84]	
			G (ALL, RNL) [85]	
			X (ALL, PXT, RNL)	
SPREC	Long-term storage	A, J, N Q, S, W [86]	B, V, C, D, E, F, G, H, I, K, T, X	P [27, 87]
				Z
BRISQ	Storage duration	< 5 years [88, 89]	5–20 years [89]	> 20 years

If predicted RNA quality is optimal according to pre-analytical factors, it is suggested to perform an integrity analysis of the total RNA through its visualization in an agarose gel and/or the calculation of the 28S:18S ratio using the RNA Integrity Number (RIN). According to recent publications, three ranges of RIN values have been set up as indicators of molecular integrity. A value greater than or equal to 7 is considered a non-degraded RNA, and therefore, it is assumed to be a high quality sample valid to carry out high-performance gene expression techniques (arrays, miRNA microarrays, RNA-Seq), and to be used in in Next Generation Sequencing (NGS) of small RNA. In contrast, RIN values between 5 and 7 are indicative of RNA slight degradation and, finally, values below 4 indicate a high level of RNA degradation. The use of samples with RIN values included in the latter two groups is not valid for high-throughput technologies for gene expression analysis. However, they may be suitable for strategies whose main objective is to detect present or absence of a particular marker, such as Endpoint PCR o miRNA detection [19–21, 90, 91].

In contrast, if a moderate RNA quality level is estimated according to pre-analytical variables, more economic analytical techniques than RIN can be performed to evaluate sample quality. A good choice could be studying transcript degradation of a housekeeping genes set by RT-qPCR (*GAPDH*, *ACTB*, *B2M*, *18S*, *ATP5E*, *TUBB*, for example) and evaluate the 3′/5′ ratio, as an indirect indicator of degradation and functionality [39, 92]. In most cases, RNA degradation is initiated by a gradual shortening of the poly(A) tail [93], which modifies the proportion of amplicons of the 3′ and 5′ region. This means that values close to 1.0 would indicate no degradation, while values further from 1.0 would indicate degradation and loss of functionality [94]. Samples with optimal quality to perform gene expression assays should present a rate of approximately 1.0 for most genes studied. Otherwise, if samples with a ratio significantly different from 1.0 are detected, they should not be considered for high performance analysis [92, 95].

Finally, if a sub-optimal quality is predicted (RIN values below 5), RIN determination itself is not a reliable measure of sample usefulness for RT-PCR or other applications, and accordingly other parameters should be taken into account in “fitness for purpose” decisions [96]. On those cases, it would be recommendable to perform endpoint PCR analyses, amplifying different fragments of several housekeeping genes, such as *G6PD*, *TBP*, *HPRT*, *ACTB*, *GAPDH* and then determine amplicon sizes by electrophoresis, loading the PCR product in an agarose gel, to start the quality control analysis. For samples showing differential size amplicons, it is assumed that whole RNA has enough quality for RT-qPCR assays. If only small amplicons are visible, it is considered that RNA has been degraded and it is only suitable for miRNAs analysis. If no amplicons are visible, the RNA quality is not enough for any gene expression study.

In summary, the expected quality of a sample and its pre-analytical variables should lead us to starting the process of quality assessment with a specific analytical technique or even a combination of them depending on the subsequent application (Fig. 1).

## A quality assessment proposal for formalin-fixed paraffin-embedded samples

**Fig. 2:**
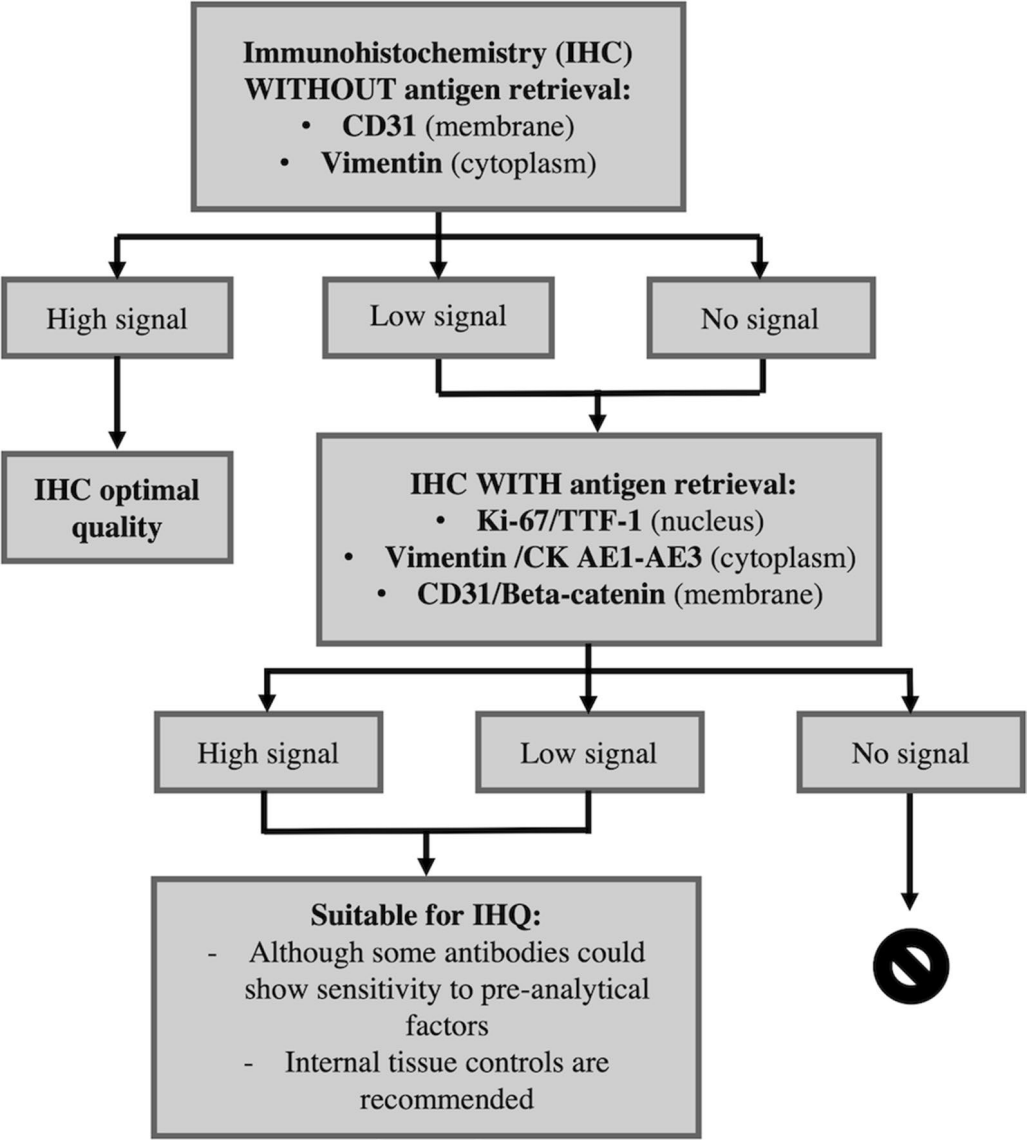
Procedures proposed to evaluate antigenicity tissue quality, in order to classify the suitability of the sample for the expected application

For the FFPE samples, according to the expected quality of the sample based on a first basic immunochemistry of CD31 and/or vimentin, a decision tree is proposed for the immunohistochemical process to be carried out (Fig. 2) in order to evaluate the antigenicity tissue quality. The antibodies selected for quality assessment were proposed based on the following criteria: (1) since they are widely used in Diagnostic Pathology routine, they could lead to an easier and rapid implementation of the quality control strategy and no changes would be necessary in work routines. Moreover, these antibodies are economically affordable and available from many reagent suppliers; (2) these antibodies are included in the Quality Assurance Program of the Spanish Society of Pathology (in Spanish: Sociedad Española de Anatomía Patológica, SEAP). This fact ensures that they are considered as antibodies used for current immunohistochemical diagnosis; (3) they hybridize with targets present in most human tissues, both healthy and pathological, which allows the quality control system to be robust.

Taking into account the above criteria, Ki-67 and TTF-1 were selected as nuclear markers, Vimentin and Cytokeratin AE1–AE3 as cytoplasmic markers; and CD31 and Beta-catenin as membrane markers. The selection of antibodies of different localizations inside cells also could help to understand how cellular location of a specific antigen can influence on its antigenicity preservation, which is currently a controversial concept.

So, in order to perform quality control on FFPE tissues a process based on two consecutives stages differentiated both by the implementation or not of an antigenic reconstitution procedure is recommended (Fig. 2). Antigenic retrieval allows recovering the antigenicity lost by the epitopes during the fixation process with formaldehyde preventing antibody recognition. The antigen retrieval process is considered as a key process for antigenicity preservation. It is advisable to use it in those samples where the concentration of the antigen to be identified is very low and in samples that have undergone prolonged periods of fixation.

We propose to carry out a first staining process with Vimentin and CD31 antibodies without antigenic reconstitution. Ki-67 is not included in this first step because it is well known that it has a low proportion of antigen and, therefore, for its proper function an antigen recovery process must be carried out. Those samples presenting high signal with Vimentin and CD31 stain, both in number of stained cells and in average intensity, would be considered as samples with optimal quality for carrying out immunohistochemistry (IHC) experiments. On the contrary, slides with low or no signal are recommended to be considered as samples of unknown quality.

Meanwhile, to evaluate the quality of those samples with unknown standards, it is proposed to carry out the second phase of the process but with a previous step of antigen retrieval. The procedure involves new staining processes, identical to the one carried out previously, but also including Ki-67 antibody. Those samples presenting a high and positive stain should be considered as samples with moderate quality to use IHC. The loss of signal between stage 1 (without antigen retrieval) and stage 2 (with antigen retrieval) would be related to pre-analytical factors affecting stability and sensitivity of epitope binding and recognition. Samples presenting no signal for the antibodies tested would have to be considered as samples of sub-optimal quality to perform IHC analysis.

## Discussion and conclusions

Human biological samples from the most prevalent chronic and rare diseases are nowadays essential for advanced biomedical research. In the case of rare diseases, only collaborative approaches make it possible to collect a relevant number of samples with high quality associated clinical data [97, 98], while it is essential for any collection that the quality of samples remains homogenous. However, the emerging lack of reproducibility of scientific results is a relevant international problem, especially in the development of clinical biomarkers for the diagnosis, treatment and follow-up of a large number of diseases [99]. Regarding tissue samples, the availability of analytical techniques to assess their quality is important and necessary to ensure reproducibility of scientific results. Fortunately, the identification of pre-analytical factors affecting integrity of samples has been very well developed in international initiatives, as SPREC, BRISQ, MIABIS, etc. Nevertheless, a standardized and extensive method to determine the usability of a sample for a particular analytical technique, or even for general tissue samples quality evaluation, has not been developed in detail. The availability of these methods, as proposed in the present work, would reduce the bias posed by a specific group of samples selected for a study. In addition, these methods would allow the identification of threshold values to determine the impact of each pre-analytical factor on the quality, integrity and functionality of tissue samples, allowing the optimization of handling, preservation and storage procedures.

Recent developments in national and international regulations on human biospecimens for research present biobanks as organizations aimed at supplying biological material with the highest quality requirements to support biomedical research [100]. In Spain, biobanks have a specific national legal regulation and normally operate under quality management systems and standardized operation procedures (SOPs) to guarantee the minimum bias among preserved tissue samples. Biobanking staff is increasingly aware of the impact that pre-analytical factors may have on the handling of tissue samples and, moreover, of the importance of having analytical tools available for taking fundamental and strategic decisions in biobanks.

In 2009, with the aim of promoting the biomedical research in Spain, a solid network of biobanks, the SBN was created to improve the overall quality of samples for research use. At present, 39 biobanks are members of the network, including regional networks of biobanks, population biobanks, disease-specific biobanks and neurological biobanks, among others. Despite being a numerous, complex and heterogeneous network, three common objectives have been established: (i) to promote the biomedical research by supplying samples with the maximum guarantee of quality; (ii) to collaborate in order to achieve the best service for the researchers; and (iii) to improve the knowledge in Biospecimen Science, in order to help on strategic decisions such as the implementation of a national quality program in biobanks. The entire network operates under a strategic plan 2018–2020, and the executive part is configured by 5 programs focused on (1) engagement of researchers and recruitment of collaborative scientific groups, (2) visibility and accessibility of the available collections and services, (3) R&D in biobanking, (4) internal and external communication, (5) specific training in biobanking procedures and network coordination. All the activity is supported by an internal structure formed by a Coordination Office, a Quality Committee, an Advisory Events Committee and an Advisory Ethical-legal Committee, headed by a coordinator advised by the Steering Committee following the recommendations of an Advisory External Scientific Committee. Similar initiatives on quality issues are faced in Europe, solved in part with the establishment of the European Research Infrastructure for Biobanking and Biomolecular Resources (BBMRI-ERIC), formed by national biobank networks, dedicated to providing researchers with the support they need to find new treatments. In all these networks, a particular concern for global quality of samples and the implementation of specific quality tests are addressed in order to improve the homogenization and standardization, and in consequence, the reproducibility of the scientific results worldwide.

To help on that issue, our Working Group has conducted thorough review of the literature and has shared common expertise between its members on a wide range of preanalytical factors and analytical tests. As a result, we have designed, two algorithms for the classification of biobank tissue samples according to their expected level of performance in various analytical procedures. Both algorithms are based on (1) a selection of preanalytical data that are relevant for the final quality of samples; and (2) on a multi-step evaluation of samples by selected analytical methods that allow a final classification in terms of expected sample quality. One of the algorithms is aimed at defining sample quality for frozen tissue samples, while a second algorithm is directed to FFPE samples.

However, the great heterogeneity of human tissue samples and the large number of pre-analytical factors associated with the quality of samples makes it very difficult to harmonize the quality criteria. Nonetheless, assessing the integrity of the tissue itself and derived biomolecules, such as its antigenicity, as the method we propose, will help to evaluate if stored human tissue samples fit for the purpose for which they were collected, as well as if they are suitable for other unspecified uses not considered previously.

To conclude, the analytical strategies and techniques that are presented here constitute a first step to evaluate the real impact of pre-analytical factors. The implementation of such analytical methods will allow the periodical evaluation of the need to perform readjustments in collection, processing and storing processes to ensure the availability of well characterized human tissue samples for their use in biomedical research.

## Abbreviations

AQUA: Automated Quantitative Analysis
BBMRI-ERIC: European Research Infrastructure for Biobanking and Biomolecular Resources
BRISQ: Biospecimen Reporting for Improved Study Quality
CIT: cold ischemia time
dHPLC: fluorescence detector-based denaturing high-performance liquid chromatography
FFPE: formalin-fixed paraffin-embedded
IHC: immunohistochemistry
LC: liquid chromatography
MS: mass spectrometry
N/A: non-available data
NGS: Next Generation Sequencing
R&D: research and development
RIN: RNA integrity number
RP: reverse phase
RPPAs: reverse phase protein array
RT-qPCR: reverse transcription quantitative polymerase chain reaction
SBN: Spanish Biobank Network
SDS-PAGE: sodium dodecyl sulphate polyacrylamide gel electrophoresis
SOPs: standardized operation procedures
SPREC: Standard PREanalytical Code
TQI: tissue quality index

## Availability of data and materials

The data supporting the conclusions of this article are included within the article.
